# Radiated glioblastoma cell-derived exosomal circ_0012381 induce M2 polarization of microglia to promote the growth of glioblastoma by CCL2/CCR2 axis

**DOI:** 10.1186/s12967-022-03607-0

**Published:** 2022-09-04

**Authors:** Chunzhi Zhang, Yuan Zhou, Ya Gao, Ze Zhu, Xianliang Zeng, Weizi Liang, Songwei Sun, Xiuli Chen, Hu Wang

**Affiliations:** 1 Department of Radiation Oncology, Tianjin Hospital, Tianjin, 300211 China; 2 Tianjin Medical University, Tianjin, 300070 China; 3 Department of Pathogenic Biology, Basic Medical College, Tianjin Medical University, Tianjin, 300070 China; 4 Department of Neuro-Surgery, Tianjin Huanhu Hospital, Tianjin, 300350 China

**Keywords:** Glioblastoma, Radiotherapy, Exosome, Microglia, M2

## Abstract

**Background:**

Radiotherapy is the primary therapeutic option for glioblastoma. Some studies proved that radiotherapy increased the release of exosomes from cells. The mechanism by which these exosomes modify the phenotype of microglia in the tumor microenvironment to further determine the fate of irradiated glioblastoma cells remains to be elucidated.

**Methods:**

We erected the co-culture system of glioblastoma cells and microglia. After radiation, we analyzing the immunophenotype of microglia and the proliferation of radiated glioblastoma cells. By whole transcriptome sequencing, we analyzed of circRNAs in exosomes from glioblastoma cells and microglia. We used some methods, which included RT-PCR, dual-luciferase reporter, et al., to identify how circ_0012381 from radiated glioblastoma cell-derived exosomes regulated the immunophenotype of microglia to further affect the proliferation of radiated glioblastoma cells.

**Results:**

Radiated glioblastoma cell-derived exosomes markedly induced M2 microglia polarization. These M2-polarized microglia promoted the proliferation of irradiated glioblastoma cells. Circ_0012381 expression was increased in the irradiated glioblastoma cells, and circ_0012381 entered the microglia via exosomes. Circ_0012381 induced M2 microglia polarization by sponging with miR-340-5p to increase ARG1 expression. M2-polarized microglia suppressed phagocytosis and promoted the growth of the irradiated glioblastoma cells by CCL2/CCR2 axis. Compared with the effects of radiotherapy alone, the inhibition of exosomes significantly inhibited the growth of irradiated glioblastoma cells in a zebrafish model.

**Conclusions:**

Our data suggested that the inhibition of exosome secretion might represent a potential therapeutic strategy to increase the efficacy of radiotherapy in patients with glioblastoma.

**Supplementary Information:**

The online version contains supplementary material available at 10.1186/s12967-022-03607-0.

## Introduction

As the highest-grade glioma, the prognosis of glioblastoma (GBM) remains dismal. Despite standard therapy, the median survival of GBM patients is approximately 15 months [1]. Growing evidence indicates that the immunophenotype of GBM significantly affects the therapeutic outcome [2–4]. The brain, the site of GBM, possesses a unique immune system with low T cell counts. Thus, GBM carries a “cold tumor” immunophenotype that is invalid for PD-1 inhibitor therapy [5, 6]. However, there is significant interest in characterizing the immune microenvironment of GBM. GBM is enriched in tumor-associated macrophages (TAMs), which are derived from tissue-resident microglia and bone marrow-derived macrophages (BMDMs), which are recruited from the peripheral circulation [5]. Landry et al. found that BMDMs were more abundant in the tumor bulk, whereas microglia dominated the tumor periphery [7]. After surgery, the residual TAMs in the GBM microenvironment are mainly microglia. A large number of studies identified that crosstalk between GBM and microglia eventually resulted in the growth of GBM and resistance to treatment [8–10]. However, the regulatory mechanisms must be further studied before translation into clinical application.

Exosomes are small extracellular vesicles (< 100 nm) responsible for the immunosuppressive effects of GBM [11]. Exosomes can communicate between cells by fusing with a target cell and unloading its contents into the cell. This has become an important means for GBM to induce the immunosuppressive phenotype of microglia [12, 13]. Mingyu et al. proved that miR-1246 in hypoxic glioma-derived exosomes induced M2 macrophage polarization [14]. Furthermore, as the main therapeutic option for GBM, radiotherapy enhances exosome biogenesis and secretion [15]. The mechanism by which these exosomes, which are induced by radiotherapy, regulate the immune phenotype of microglia remains to be clarified.

In the present study, we isolated exosomes from irradiated GBM cells and confirmed that these exosomes could be internalized by microglia. Next, we found that these exosomes could induce the M2 polarization of microglia, which further increased the proliferation of irradiated GBM cells. GW4869, which blocks exosome generation, could neutralize the M2 polarization of microglia and the proliferation of irradiated GBM cells. By sequencing, we found that circular RNAs (circRNAs) were differentially expressed. Among them, circ_0012381 only existed in irradiated GBM cells and the microglia that phagocytized the exosomes from irradiated GBM cells. Furthermore, circ_0012381 induced M2 microglia polarization by sponging with miR-340-5p to increase ARG1 expression. M2-polarized microglia reduced phagocytosis and promoted the irradiated GBM growth by CCL2/CCR2 axis. Furthermore, it was proved that C/EBPB, which is induced by radiation, co-transcriptionally regulated the level of circ_0012381 and CCR2 in GBM cells. Finally, compared with the effects of radiotherapy alone, the inhibition of exosomes significantly inhibited the growth of irradiated GBM cells in vivo.

## Materials and methods

### Cell culture and exosome isolation

U251 and U87 human glioblastoma cells and HMC3 human microglia were obtained from the Institute of Biochemistry and Cell Biology, Chinese Academic of Science. All cell lines were grown in Dulbecco’s modified Eagle’s medium (Gibco, USA) supplemented with 10% exosome-depleted fetal bovine serum (FBS, Gibco), 2 mM glutamine (Sigma-Aldrich, USA), 100 units/ml penicillin (Sigma-Aldrich), and 100 µg/ml streptomycin (Sigma-Aldrich); incubated at 37 °C in 5% CO_2_; and sub-cultured every 2–3 days. Exosomes were isolated from cell culture supernatant as previously described for subsequent analysis [16]. In brief, the collected medium was centrifuged at 300*g* (10 min) and 2000*g* (10 min) at 4 °C. Then, the supernatant was centrifuged at 10,000*g* for 10 min at 4 °C. Finally, the supernatant was centrifuged at 110,000*g* for 2 h at 4 °C to obtain exosomes. Exosomes were then re-suspended in pre-cooled PBS.

### Irradiation and chemistry treatment

Irradiation was performed in a linear accelerator (Varian 600, Varian, USA) at a dose rate of 3.2 Gy/min. circ_0012381 overexpression, circ_0012381 inhibitor, circRNA control, miR-340-5p overexpression, miR-340-5p inhibitor, and miRNA control lentiviruses were synthesized by Genechem (Shanghai, China) (Additional file 1: Table S1). To block exosome generation, U251 or U87 cells were incubated with 10 µM GW4869 (Sigma-Aldrich), which was initially dissolved in DMSO into a stock solution of 5 mM and diluted in culture medium, for 24 h.

### Scanning electron microscopy

The isolated exosomes suspended in phosphate-buffered saline (PBS) were adhered to Formvar-coated copper grids. The preparations were attached to metal stubs and coated with gold to a thickness of 15 nm. The morphology and diameter of the exosomes were analyzed using a Philips XL30 scanning electron microscope.

### Nanoparticle tracking analysis (NTA)

Analysis of the absolute size distribution of the exosomes was performed using NanoSight NS300 (Malvern, UK). The light scattered by the exosomes following laser illumination was captured by a camera, and a video file of exosomes moving under Brownian motion was created. NTA software tracked and individually analyzed particles of 10–1000 nm in size. Three recordings were performed for each sample.

### RNA extraction and qRT-PCR

Exosome RNA extraction was conducted using the SeraMir^™^ Exosome RNA Extraction Kit (System Biosciences, USA) after isolating exosomes using Exoquick (System Biosciences).

TRIzol was used to extract total cell RNA according to the manufacturer’s protocol. Reverse transcription and PCR were performed as previously described [17]. All primers are presented in Additional file 1: Table S2.

### Phagocytosis assay

Fluorescent-labeled polystyrene microparticles (0.99 µm; Polysciences, 10 µl/tube) were coated with FBS) diluted to 50% in PBS and incubated with HMC3 cells (1×10^6^/tube) for 30 min at 37 °C. The reaction was stopped by adding ice-cold PBS. At the end of the incubation, the remaining beads were gently washed off the cells and fixed with 4% PFA at room temperature. After staining with Hoechst 33342 at room temperature for 15 min, cells were washed and analyzed by fluorescence microscopy (Nikon E400, Lewisville, TX, USA) for internalized particles.

### RNA sequencing, bioinformatics analysis, and luciferase reporter assay

Total RNA was extracted from exosomes and HMC3 cells by Trizol reagent (Invitrogen). Sequencing libraries were generated using NEBNext^®^Multiplex Small RNA Library Prep Set for Illumina^®^(NEB, USA) following manufacturer’s recommendations. Paired-end sequencing was performed using the Illumina HiSeq 4000 platform. All RNA-seq and bioinformatic analysis were performed at Novogene Co., Ltd (Beijing, China). Expression data were normalized to the number of fragments per kilobase of transcript per million fragments mapped, and the differentially expressed genes were identified by the DESeq2 package of R software using default values. Differentially expressed genes were defined by |log2FC|> 0.5 and adjusted p-value < 0.05. RegRNA 2.0 (http://regrna2.mbc.nctu.edu.tw/index.html) was used to predict the binding sites between circ_0012381 and miR-340-5p. circ_0012381 wild-type and mutant reporter vectors were constructed and inserted into the pGL3 plasmid (Genechem, China). TargetScan (http://www.targetscan.org) was used to predict the targets of miR-340-5p. Reporter genes carrying wild-type and mutant pGL3-ARG1-3′UTR (Genechem, China) were synthesized. HMC3 cells were co-transfected with luciferase reporters and miR-340-5p mimic. Two days after transfection, the reporter gene activities were measured using a dual-luciferase reporter assay kit (Promega, USA) according to the manufacturer’s instructions.

### Cell counting Kit-8 (CCK8) assay)

The CCK8 assay was conducted in accordance with the manufacturer’s instructions (WST-8, Dojindi Labs, Kumamoto, Japan). In brief, U251 and U87 cells were seeded at a density of 3000 cells/well in a 96-well plate and treated with ethanol. After 24, 48, or 72 h, 10 µl of CCK8 solution were added to each well of the plate, and the wells were incubated for 2 h. Absorbance at 450 nm was evaluated using a Multiscan MS spectrophotometer (Labsystems, Stockholm, Sweden).

### Immunohistochemistry of tumor samples

Clinical samples were collected from patients with GBM who underwent surgery in the Department of Neurosurgery, Tianjin Huanhu Hospital. Paraffin-embedded tissue sections were used to examine CD68 and CD163 expression in excised tumors. Immunohistochemistry was performed as described previously [17].

### Western blotting

Western blotting was performed as described previously [17]. Primary antibodies include ARG1, Beclin1, C/EBPB, CCR2, GAPDH (1: 1000 dilution, Santa Cruz, USA).

### Zebrafish xenograft model

Before injection, U251 and U87 cells were labeled with LV3 (Genechem), and HMC3 cells were labeled with LV10 (Genechem). The cell transplantation protocol was modified from the protocol described by Geiger et al. [18]. Needles were pulled using a P-97 Flaming/Brown Micropipette Puller (Sutter Instrument Co.). A Nanoject II microinjector (Drummond Scientific) was used to transplant approximately 200 cells (GBM cells: HMC3 cells = 2:1) into the center of the embryonic yolk sac. After injection, embryos were maintained for 1 h at 28 °C before incubation at 31 °C. Five days after injection, zebrafish larvae with similar sizes of transplanted cells were collected, exposed to 6 Gy of radiation, and then cultured at 34 °C until the end of the experiments. The size of xenograft tumors in zebrafish was examined by fluorescence microscopy. Imaging was performed by stereomicroscopy (MVX10, Olympus, Japan).

### Chromatin immunoprecipitation assay

Chromatin was prepared by the Millipore/Upstate ChIP assay kit (Millipore) according to the manufacturer’s instruction. The ChIP assay was performed as described previously [17].

### Data analysis and statistics

GraphPad Prism software version 8.0 (GraphPad Software, San Diego, CA, USA) was used to build graphs and diagrams and perform statistical analysis. Data are presented as means with standard error bars.

## Results

### Irradiated GBM cell-derived exosomes reduced their phagocytic capacity by inducing the M2 polarization of microglia

**Fig. 1:**
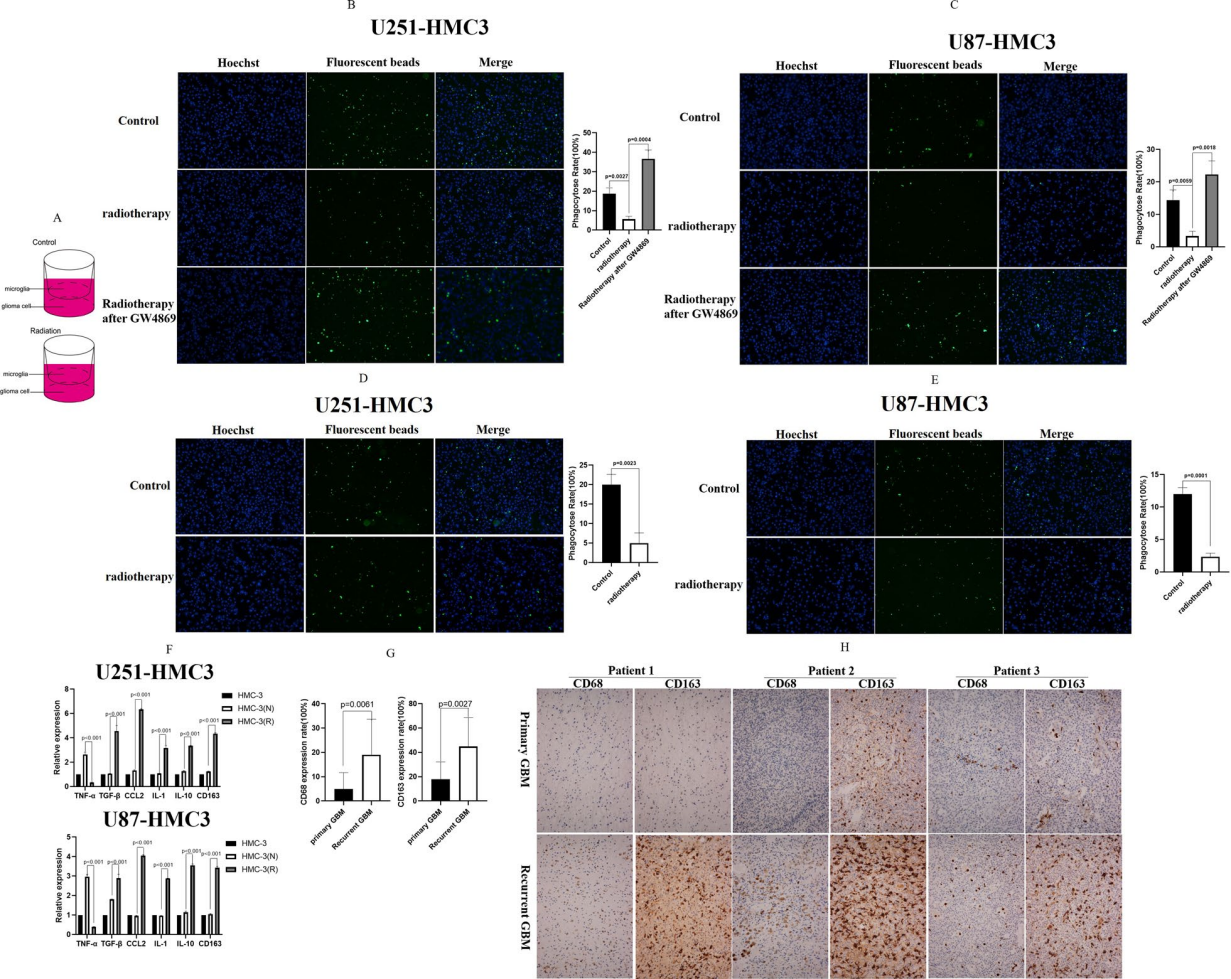
Irradiated GBM cell-derived exosomes reduced their phagocytic capacity by inducing the M2 polarization of microglia. **A** The sketch map showed that GBM and HMC-3 were implanted in the co-culture system. **B** A phagocytosis assay proved that 2 Gy irradiation significantly decreased the phagocytic capability of HMC3 cells in the U251–HMC3 co-culture system. **C** A phagocytosis assay proved that 2 Gy irradiation significantly decreased the phagocytic capability of HMC3 cells in the U87–HMC3 co-culture system. **D** Exosomes from irradiated U251 cells significantly decreased the phagocytic capability of HMC3 cells. **E** Exosomes from irradiated U87 cells significantly decreased the phagocytic capability of HMC3 cells. **F** RT-PCR demonstrated that TGFβ, CCL2, IL-1, IL-10, and CD163 expression increased in HMC3 cells that phagocytized exosomes from irradiated GBM cells, whereas TNFα expression decreased. The opposite results were observed in HMC3 cells that phagocytized exosomes from non-irradiated GBM cells. **G** In 12 pairs of GBM tissues including primary and recurrent GBM, immunohistochemistry demonstrated that the expression of CD163, which is a marker of M2 polarization, significantly increased in recurrent GBM. **H** Three representative pathological cases

In addition to the adaptive immune checkpoints, the innate immune checkpoints, which are involved in detecting and clearing malignancy via phagocytosis, also play important roles in tumor-mediated immune escape [19]. Gregor et al. reported that GBM escaped innate immunosurveillance by disturbing the phagocytic effect between GBM cells and microglia [20]. However, the mechanism by which radiotherapy alters the phagocytic effect between GBM cells and microglia has not been reported. We establish a co-culture system of GBM cells and microglia to simulate the actual clinical situation. As presented in Fig. 1A, we plated U251/U87 cells and HMC-3 cells in the co-culture system. A phagocytosis assay proved that irradiation (2 Gy) significantly decreased the phagocytic capability of microglia in the co-culture system (Fig. 1B, C). Exosomes are involved in several biological processes and diseases, such as immune responses and cancer progression. By delivering proteins, metabolites, and nucleic acids into recipient cells, exosomes effectively alter the biological responses of their targets. We used GW4869 to inhibit exosome secretion by irradiated GBM cells. Compared with the microglia in the radiated co-culture system, a phagocytosis assay demonstrated that the phagocytic capability of microglia was significantly recovered by using GW4869 in the co-culture system after radiation. Subsequently, we extracted exosomes from the culture medium to culture HMC3 cells. Compared with the effects of exosomes obtained from non-irradiated GBM cells, exosomes from irradiated GBM cells significantly decreased the phagocytic capability of miFcroglia (Fig. 1D, E). These results proved that irradiated GBM cell-derived exosomes reduced the phagocytic capacity of microglia in the co-culture system.

Furthermore, we analyzed the alteration of the microglia immunophenotype after co-culture with exosomes to delineate how irradiated GBM cell-derived exosomes reduced the phagocytic capacity of microglia. In the tumor, microglia can polarize into two different phenotypes, namely the classic M1 phenotype and the alterative M2 phenotype. M1 microglia can eliminate tumor cells, whereas M2 microglia promote the growth of tumor cells [21]. The confocal microscopy revealed that microglia phagocytized the exosomes (Additional file 1: Fig. S1). According to the literature [22], we checked the mRNA expression of cytokines and chemokines to discriminate M2 microglia from M1 microglia. The results illustrated that TGFβ, CCL2, IL-1, IL-10 and CD163 expression was increased in microglia that phagocytized exosomes from irradiated GBM cells, whereas TNFα expression was decreased (Fig. 1F). The opposite result was observed for microglia that phagocytized exosomes from non-irradiated GBM cells (Fig. 1F). These results indicated that exosomes from irradiated GBM cells induced the M2 polarization of microglia. To further verify this result, we analyzed 12 pairs of GBM tissues including primary and recurrent GBM. On immunohistochemistry, the expression of CD163, which is a marker of M2 polarization, significantly increased in recurrent GBM (Fig. 1G). Figure 1H presented three representative pathological cases. Taken together, these results illustrated that irradiated GBM-derived exosomes reduced their phagocytic capacity by inducing the M2 polarization of microglia.

### M2-polarized microglia promoted the proliferation of irradiated GBM cells by CCL2/CCR2

**Fig. 2:**
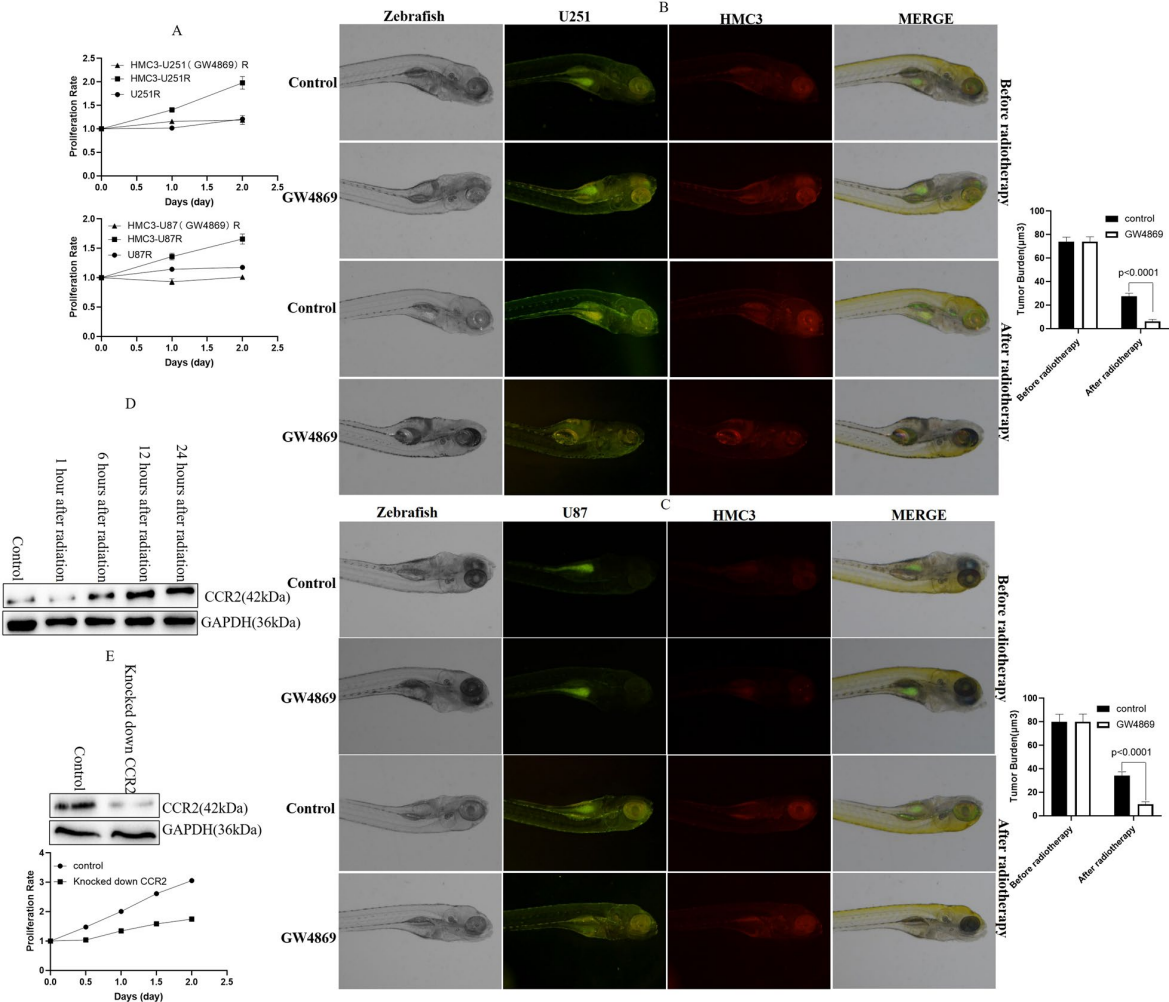
M2-polarized microglia promoted the proliferation of irradiated GBM cells by CCL2/CCR2. **A** M2-polarized HMC3 cells obviously promoted the proliferation of irradiated GBM cells. **B** GW4869 suppressed the growth of irradiated U251 cells and increased the efficacy of radiotherapy in the co-culture system of U251 and HMC3 cells in a zebrafish model. **C** GW4869 suppressed the growth of irradiated U87 cells and increased the efficacy of radiotherapy in a co-culture system of U87 and HMC3 cells in a zebrafish model. **D** The expression of CCR2 increased in U251 cells after radiation. **E** Knocked down CCR2 significantly relieved the proliferation of the radiated U251 cells by M2 HMC3 cells inducing

Next, we assessed how M2-polarized microglia regulated the proliferation of irradiated GBM cells. Compared with irradiated GBM cells under non-coculture conditions, M2-polarized microglia obviously promoted the proliferation of irradiated GBM cells in vitro (Fig. 2A). GW4869, which blocks exosome generation, could neutralize the M2 polarization of microglia and the proliferation of irradiated GBM cells. Our result is similar to those of prior studies in which M2 microglia promoted the proliferation of glioma cells [23, 24]. Subsequently, we used a zebrafish model to verify the result in vivo. After co-culture with HMC3 cells, U251 or U87 cells treated with GW4869 were implanted in zebrafish embryos and exposed to 6 Gy of radiation on the fifth day after implantation, at which time the tumor size was approximately 77 µm^3^ (the average size was 74 µm^3^ in the U251 zebrafish model and 80 µm^3^ in the U87 zebrafish model). Ten days after implantation, both U251 and U87 tumors, which were treated by GW4869, displayed the reduced growth (Fig. 2B, C). These data identified that M2-polarized microglia promoted the proliferation of irradiated GBM cells in vivo and in vitro. A previous study suggested that CCL2/CCR2 plays an important role in the proliferation of glioma cells [25]. Our above data have confirmed that the level of CCL2 increased in M2 microglia. By Western blot, we found that the expression of CCR2 increased in U251 cells after radiation (Fig. 2D). So, we inferred that M2-polarized microglia promoted the proliferation of irradiated GBM cells by CCL2/CCR2. Furthermore, we identified that knocked down CCR2 significantly relieved the proliferation of the radiated U251 cells by M2 HMC3 cells inducing (Fig. 2E). To sum up, these data proved that M2-polarized microglia promoted the proliferation of irradiated GBM cells by CCL2/CCR2. Furthermore, these results also demonstrated that the inhibition of exosome secretion suppressed GBM growth and increased the efficacy of radiotherapy in a co-culture system of GBM cells and microglia.

### Circ_0012381 from irradiated GBM cell-derived exosomes induced the M2 polarization of microglia and inhibited phagocytosis

**Fig. 3:**
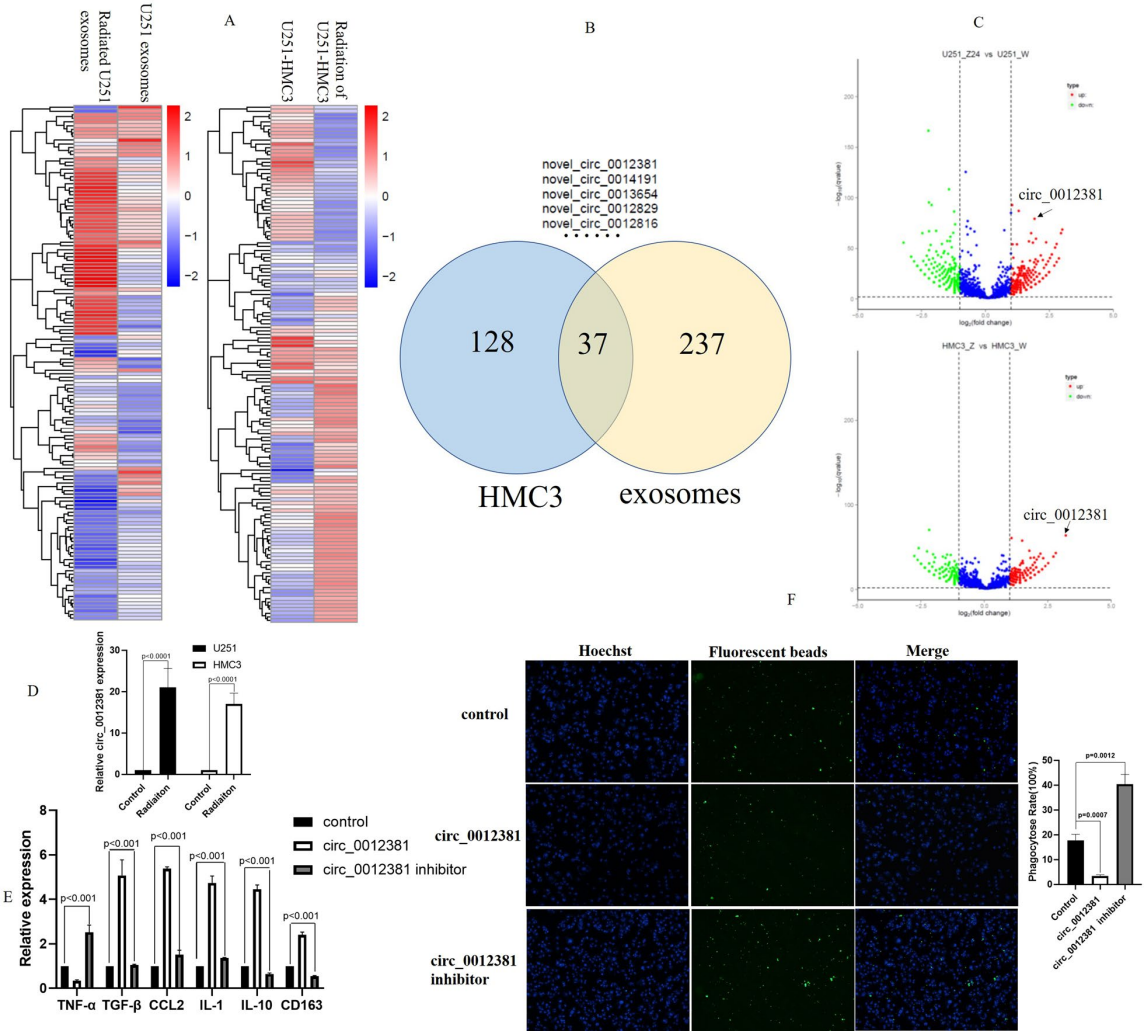
circ_0012381 from irradiated GBM cell-derived exosomes induced the M2 polarization of microglia and inhibited phagocytosis. **A** The massive circRNAs were significantly different in exosomes secreted by irradiated GBM cells compared with exosomes secreted by non-irradiated GBM cells. As well, compared with HMC3 cells that phagocytized exosomes secreted by non-irradiated GBM cells, a lot of circRNAs were significantly different in HMC3 cells which phagocytized exosomes from irradiated GBM cells. **B** The number of the overlapping upregulated circRNAs was 37 in 2 heat maps. **C** Upregulation of circ_0012381 was the most significantly in 37 circRNAs. **D** RT-PCR identified that radiation upregulated the level of circ_0012381 of U251 and HMC3 in co-cultuer system. **E** Circ_0012381 increased TGFβ, CCL2, IL-1, IL-10, and CD163 expression and decreased TNFα expression in HMC3 cells. **F** Circ_0012381 inhibited the phagocytic capability of HMC3 cells

It has been reported that radiotherapy could increase the number of exosomes secreted by cells [26]. We isolated exosomes from non-irradiated and irradiated GBM cells. Transmission electron microscopy illustrated that the exosomes were rounded particles of approximately 80–100 nm in size with a double-layer membrane (Additional file 1: Fig. S2A, B). As previously reported, NTA demonstrated that radiotherapy increased the number of exosomes secreted by U251 and U87 cells (Additional file 1: Fig. S2C). In addition to the number of exosomes, recent studies also proved that radiation induced changes in the composition of exosomes released by cells [26]. To clarify the mechanism by which irradiated GBM cell-derived exosomes induced immunophenotypic changes in microglia, we performed whole transcriptome sequencing of exosomes and HMC3 cells. Figure 3A showed that the massive circRNAs were significantly different in exosomes secreted by irradiated GBM cells compared with exosomes secreted by non-irradiated GBM cells. As well, compared with HMC3 cells that phagocytized exosomes secreted by non-irradiated GBM cells, a lot of circRNAs were significantly different in HMC3 cells which phagocytized exosomes from irradiated GBM cells (Fig. 3A). The number of the overlapping upregulated circRNAs was 37 in 2 heat maps (Fig. 3B). In these circRNAs, upregulation of circ_0012381 was the most significantly (Fig. 3C). It is proved by RT-PCR in U251-HMC3 co-culture system (Fig. 3D). To further determine whether circ_0012381 induce M2 polarization in HMC3 cells, we checked the mRNA expression of cytokines and chemokines in HMC3 cells. Figure 3E illustrated that circ_0012381 increased TGFβ, CCL2, IL-1, IL-10, and CD163 expression and decreased TNFα expression in HMC3 cells. These findings directly proved that circ_0012381 induced the M2 polarization of HMC3 cells. Furthermore, we performed a phagocytic assay. The result indicated that circ_0012381 inhibited the phagocytic activity of HMC3 cells (Fig. 3F). Together, these results revealed that circ_0012381 in exosomes from irradiated GBM cells induced the M2 polarization of microglia and inhibited phagocytosis.

### Circ_0012381 sponged with miR-340-5p to induce the M2 polarization of microglia

**Fig. 4:**
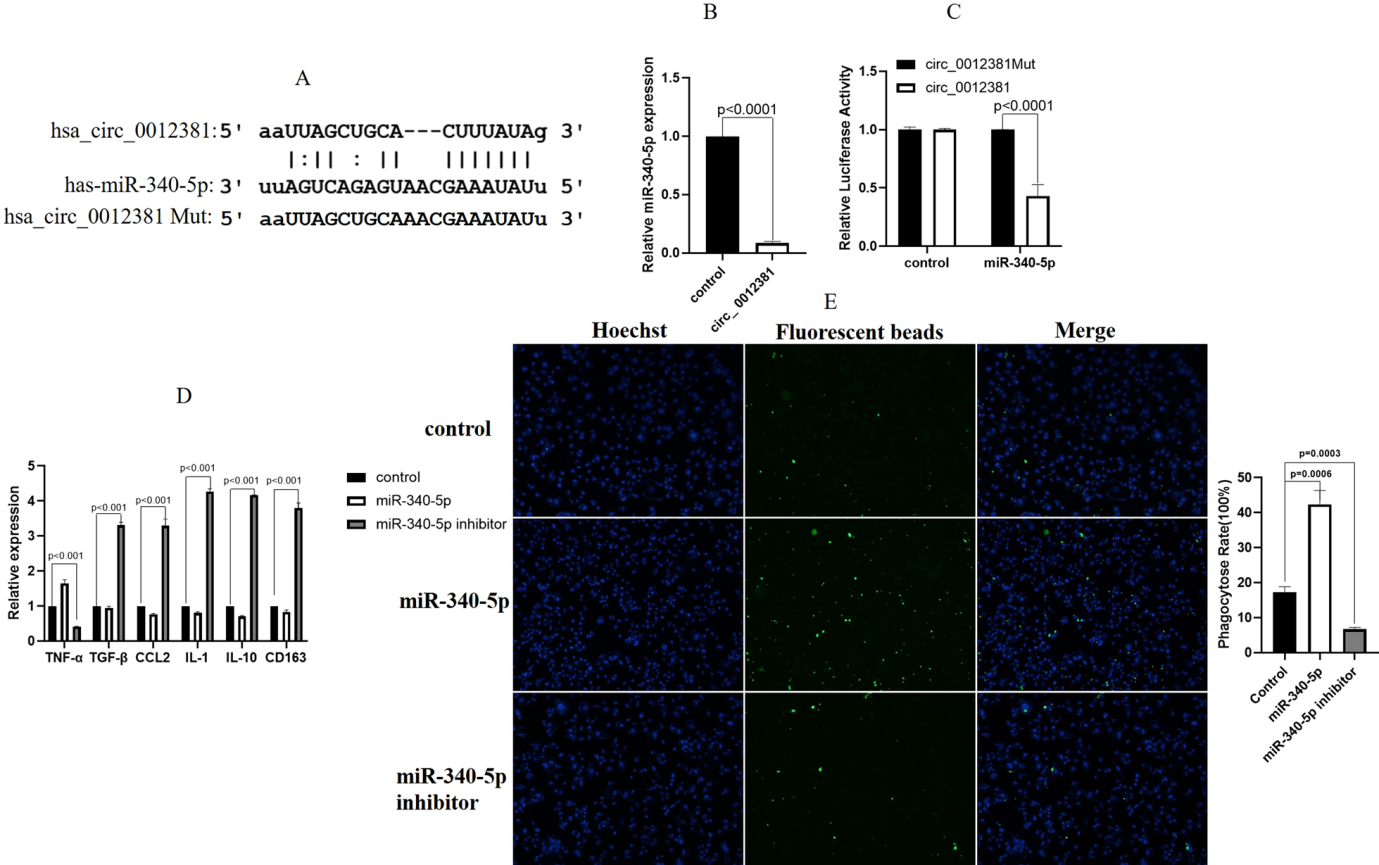
circ_0012381 sponged with miR-340-5p to induce the M2 polarization of microglia. **A** RegRNA 2.0 predicted that circ_0012381 contained a conserved binding site for miR-340-5p. **B** RT-PCR revealed that circ_0012381 expression was negatively correlated with miR-340-5p expression. **C** A dual-luciferase reporter assay indicated that miR-340-5p markedly decreased the luciferase activity of wild-type circ_0012381. **D** miR-340-5p induced the M2 polarization of HMC3 cells. **E** miR-340-5p inhibited the phagocytic capability of HMC3 cells

Recent studies demonstrated that circRNAs were involved in miRNA inhibition [27]. Concerning the molecular mechanism of circ_0012381, we found that circ_0012381 potentially sponged with miR-340-5p using RegRNA2.0(http://regrna2.mbc.nctu.edu.tw/index.html). Moreover, circ_0012381 contains a conserved binding site for miR-340-5p (Fig. 4A). RT-PCR indicated that circ_0012381 expression was negatively correlated with miR-340-5p expression (Fig. 4B). We performed a dual-luciferase reporter assay to validate the sponging relationship between circ_0012381 and miR-340-5p in U251 cells. The result demonstrated that miR-340-5p markedly decreased the luciferase activity of wild-type circ_0012381 (Fig. 4C). To further determine whether miR-340-5p induce the M2 polarization of HMC3 cells, we examined the mRNA expression of cytokines and chemokines. Figure 4D revealed that miR-340-5p induced the M2 polarization of HMC3 cells. Furthermore, Fig. 4E demonstrated that miR-340-5p inhibited the phagocytic capability of HMC3 cells. Together, these results indicated that circ_0012381 induced the M2 polarization of microglia and inhibited phagocytosis by sponging with miR-340-5p.

### MiR-340-5p targeted ARG1 to inhibit the M2 polarization of microglia

**Fig. 5:**
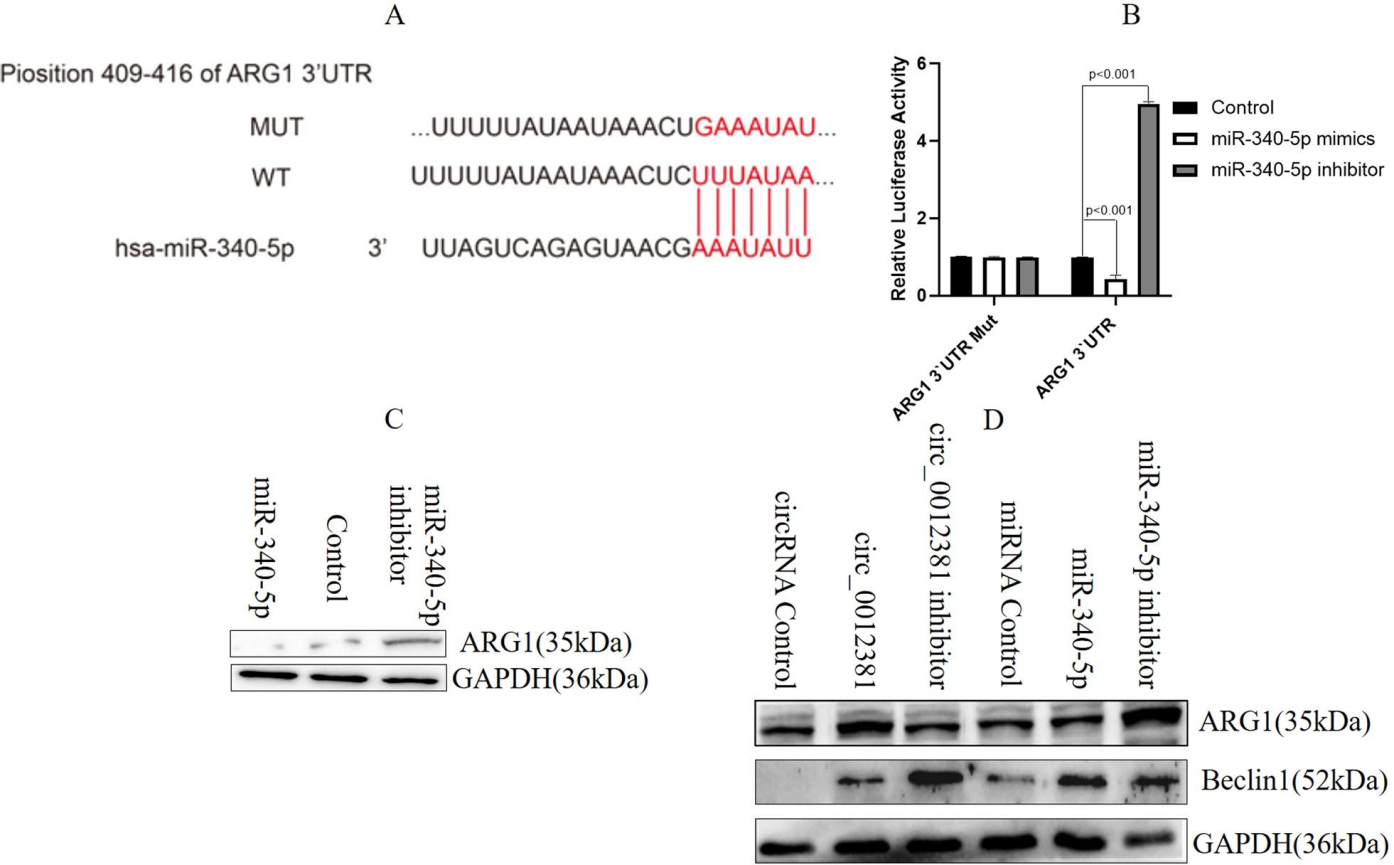
MiR-340-5p targeted ARG1 to inhibit the M2 polarization of microglia. **A** TargetScan predicted that ARG1 was a potential target gene of miR-340-5p. **B** The luciferase reporter assay revealed that miR-340-5p inhibitor significantly enhanced the activity of the wild-type reporter vector, whereas miR-340-5p mimic decreased its luciferase activity. **C** Western blot illustrated that ARG1 expression was upregulated in HMC3 cells transfected with the miR-340-5p inhibitor lentivirus and downregulated in HMC3 cells transfected with the miR-340-5p overexpression lentivirus compared with the control value. **D** Western blot illustrated circ_0012381 and miR-340-5p inhibitor significantly upregulated ARG1 expression. Moreover, circ_0012381 and miR-340-5p inhibitor significantly downregulated beclin 1 expression

To identify the target of miR-340-5p, which is involved in regulating the immunophenotype of HMC3 cells, we used TargetScan (http://www.targetscan.org/vert_72/). The result identified ARG1 as a potential target gene of miR-340-5p (Fig. 5A). To examine whether miR-340-5p directly regulates ARG1, we generated pGL3-ARG1-3′UTR wild-type and pGL3-ARG1-3′UTR mutation luciferase reporter vectors. The luciferase reporter assay revealed that miR-340-5p inhibitor significantly enhanced the activity of the wild-type reporter vector, whereas miR-340-5p mimic decreased its luciferase activity (Fig. 5B). Furthermore, western blotting demonstrated that ARG1 expression was upregulated in HMC3 cells transfected with the miR-340-5p inhibitor lentivirus and downregulated in HMC3 cells transfected with the miR-340-5p overexpression lentivirus compared with the finding in the control (Fig. 5C). To further uncover whether circ_0012381 induces the M2 polarization of HMC3 cells by sponging with miR-340-5p, we checked the expression of the M2 marker ARG1 [28, 29]. The result illustrated that circ_0012381 and miR-340-5p inhibitor significantly upregulated ARG1 expression (Fig. 5D). Moreover, circ_0012381 and miR-340-5p inhibitor significantly downregulated the expression of beclin 1 (Fig. 5D), which promotes the phagocytosis of microglia [30]. The results indirectly proved that circ_0012381 and miR-340-5p inhibitor promoted M2 polarization and inhibited the phagocytic capability of HMC3 cells. Together, the aforementioned results highlighted that miR-340-5p, which was sponged by circ_0012381, inhibited the M2 polarization of microglia and enhanced their phagocytic capability by targeting ARG1.

### The radiation induced C/EBPB to increase the level of circ_0012381 and CCR2 in glioma cells

**Fig. 6:**
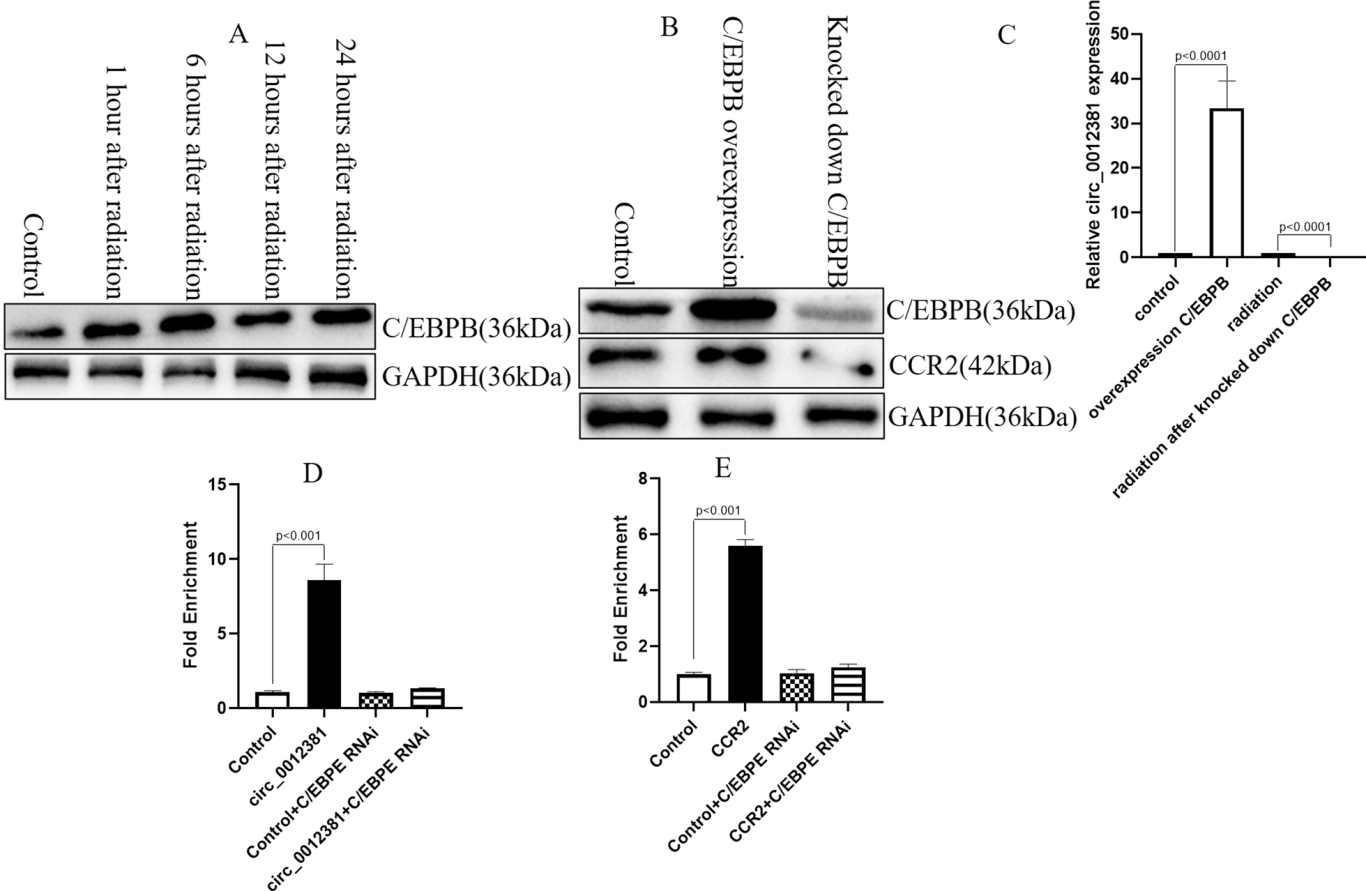
The radiation induced C/EBPB to increase the level of circ_0012381 and CCR2 in glioma cells. **A** Radiation induced the expression of C/EBPB in U251 cells. **B** The expression of CCR2 decreased after knocked down C/EBPB. **C** The level of circ_0012381 decreased after knocked down C/EBPB. **D** ChIP assay identified that C/EBPB transcriptionally regulated the level of circ_0012381 in U251 cells. **E** ChIP assay identified that C/EBPB transcriptionally regulated the expression of CCR2 in U251 cells

For further understand how circ_0012381 and CCR2 are induced by radiation, we used PROMO database (http://alggen.lsi.upc.es/cgi-bin/promo_v3/promo/promoinit.cgi?dirDB=TF_8.3) to check the transcriptional factor of circ_0012381 and CCR2. We surprisedly found that C/EBPB co-transcriptionally regulated the level of circ_0012381 and CCR2 in glioma cells. By Western Blot, we identified that radiation induced the expression of C/EBPB in U251 cells (Fig. 6A). After knocked down C/EBPB, it was identified that the level of circ_0012381 and CCR2 decreased (Fig. 6B, C). For further assessing the transcriptional role of C/EBPB in circ_0012381 and CCR2, we measured the binding of C/EBPB to the promoter of circ_0012381 and CCR2 in U251 cells by ChIP. The results showed that the binding of C/EBPB were remarkably enriched in the promoter of circ_0012381 and CCR2 compared with the negative control region, and no relevant chromatin enrichment were found in knocked down C/EBPB cells (Fig. 6D, E). Taken together, these data identified that radiation-induced C/EBPB transcriptionally regulated the level of circ_0012381 and CCR2 in GBM cells.

## Discussion

Radiotherapy, which is an important component of the standard treatment of GBM, provides survival benefits in GBM patients. However, patients with GBM usually experience tumor recurrence at the resection margin, at which the highest radiation doses are delivered [31]. Moreover, studies proved that radiotherapy can modulate the tumor immune microenvironment by triggering the release of pro-inflammatory and anti-inflammatory mediators, increasing the counts of tumor-infiltrating immunostimulatory cells and immunoinhibitory cells, and enhancing the expression of neoantigens [32, 33]. However, there is a lack of research on radiation-induced immunophenotypic changes in immune cells. Our study first identified that irradiated GBM cells induced the M2 polarization of microglia via exosomes. Subsequently, these M2-polarized microglia promoted the proliferation of irradiated GBM cells.

Exosomes, which are released by different types of tumor cells, carry discrete molecules, including mRNAs, miRNAs, lincRNAs, circRNAs, and proteins, and play an important role in cell-to-cell communication. Their function mainly focuses on altering the phenotype of recipient cells through releasing various contents [34]. Excellent reviews have discussed that exosomal non-coding RNAs play important roles in GBM [35]. As a comprehensive class of RNAs generated by non-linear back-splicing, circRNAs have been widely studied in GBM [36]. They perform their function by acting as miRNA sponges, binding with proteins, and undergoing translation. Furthermore, some studies illustrated that circRNAs participated in cell-to-cell communication via exosomes [37].

**Fig. 7:**
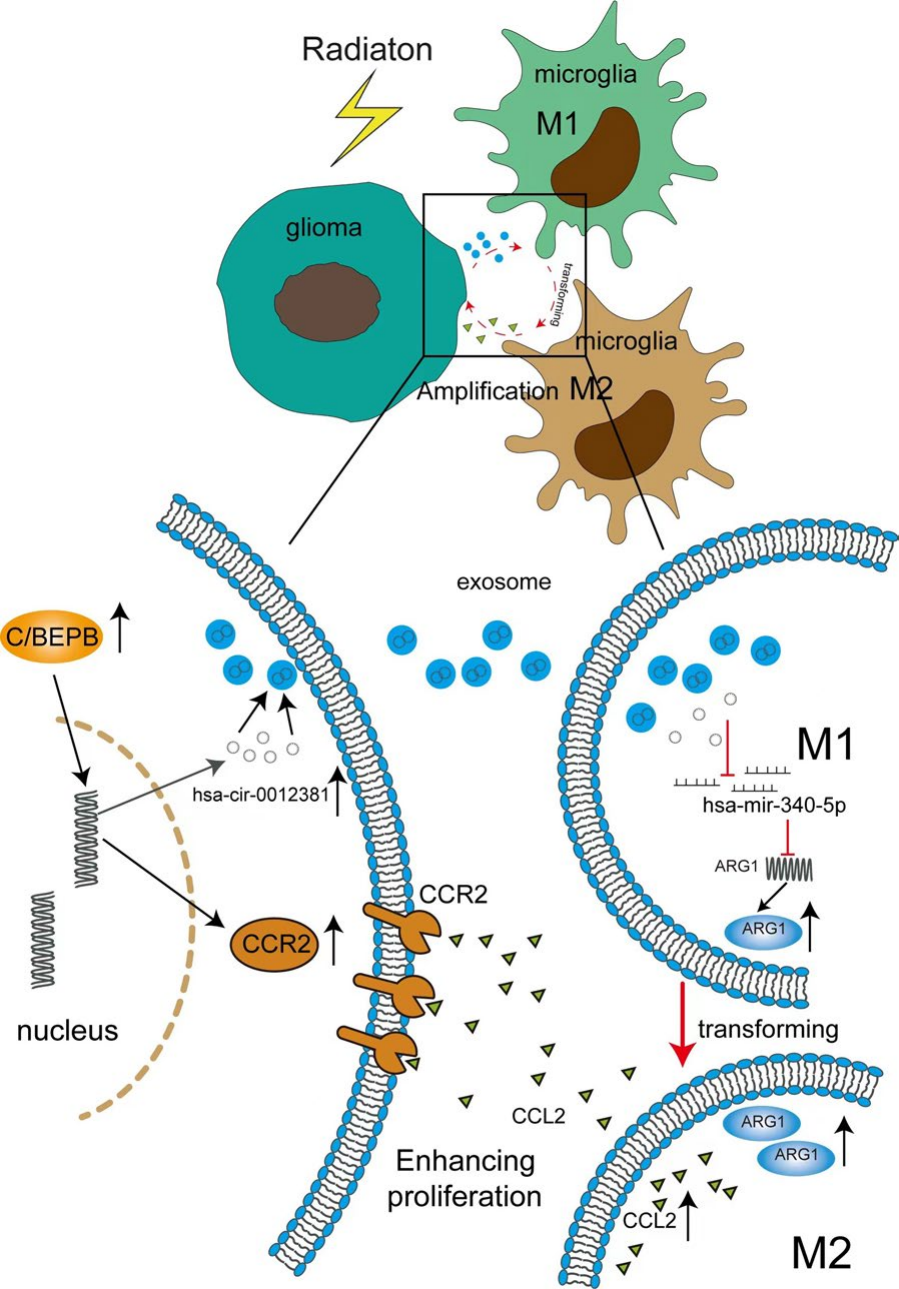
The skeleton diagram on that radiation induces the interaction between glioma cells and microglia to promote the proliferation of the radiated glioma cells

In our study, we studied how radiotherapy initiated the interaction between GBM cells and microglia to further determine the fate of irradiated GBM cells. We firstly found that the radiated GBM cells-derived exosomes induced the M2 polarization of microglia. The phagocytic capacity of M2-polarized microglia was decreased. Furthermore, these M2-polarized microglia promoted the proliferation of irradiated GBM cells. We used GW4869, which blocks the generation of exosomes, to neutralize the M2 polarization of microglia and inhibit the proliferation of irradiated GBM cells in vitro and in vivo. Mechanically, we performed whole transcriptome sequencing using exosomes and HMC3 cells. The result indicated that circ_0012381 expression increased in irradiated GBM cells and that circ_0012381 entered microglia via exosomes. Using RegRNA 2.0, we found that circ_0012381 potentially sponged with miR-340-5p. This was confirmed by a luciferase assay. Subsequently, we identified ARG1 as a potential target gene of miR-340-5p using TargetScan. The conjecture has been also proved. Furthermore, we found that irradiation-induced C/EBPB co-transcriptionally regulated the level of circ_0012381 and CCR2. So, as shown in Fig. 7, irradiation-induced C/EBPB transcriptionally regulated the level of circ_0012381 and CCR2. circ_0012381 was delivered to microglia by exosomes and induced the M2 polarization of microglia by sponging with miR-340-5p, which targets ARG1. M2 microglia secreted CCL2 to promote the proliferation of irradiated GBM cells by CCL2/CCR2 axis.

In summary, our data demonstrated that irradiated glioblastoma cell-derived exosomes promoted the M2 polarization of microglia and decreased their phagocytic capability. By secreting CCL2, M2 microglia further promoted the proliferation of irradiated GBM cells by binding with CCR2. Mechanically, circ_0012381 plays an important role in regulating the M2 polarization of microglia. However, as shown in Fig. 3A, the other circRNAs were only detected in exosomes secreted by irradiated GBM cells except for circ_0012381. We only study circ_0012381 because the level of circ_0012381 was significantly higher in HMC3 cells which phagocytized exosomes from irradiated GBM cells than in HMC3 cells that phagocytized exosomes secreted by non-irradiated GBM cells. The other circRNAs, which were only detected in exosomes secreted by irradiated GBM cells, may also be involved in regulating the M2 polarization of microglia. So, the inhibition of exosome secretion from irradiated GBM cells seems more reasonable in blocking the interaction between GBM cells and microglia to inhibit the proliferation of irradiated GBM cells. These are reflected in vivo and in vitro. Thus, our data suggested that the inhibition of exosome secretion was a potential therapeutic strategy for increasing the efficacy of radiotherapy in patients with GBM.

## Supplementary Information

**Additional file 1: Figure S1.** The confocal microscopy revealed that HMC3 cells phagocytized exosomes released by U251 and U87 cells. **Figure S2. **Radiotherapy increased the number of exosomes secreted by glioblastoma multiforme (GBM) cells.** A** Transmission electron microscopy demonstrated that the secreted exosomes were rounded particles with a double-layer membrane. **B** The size of these exosomes was approximately 80–100 nm. **C** Nanoparticle tracking analysis indicated that radiotherapy increased the number of exosomes secreted by U251 and U87 cells. **Table S1** All oligonucleotide sequences and **Table S2.** All primers of RT-PCR.

## Data Availability

Publicly available datasets were analyzed in this study. This data can be found below: RegRNA 2.0 (http://regrna2.mbc.nctu.edu.tw/index.html); TargetScan (http://www.targetscan.org/vert_72/); PROMO database (http://alggen.lsi.upc.es/cgi-bin/promo_v3/promo/promoinit.cgi?dirDB=TF_8.3).
