# Supplementary material for: Radiated glioblastoma cell-derived exosomal circ_0012381 induce M2 polarization of microglia to promote the growth of glioblastoma by CCL2/CCR2 axis
Source: J Transl Med. 2022 Sep 4;20:388. doi: 10.1186/s12967-022-03607-0 (PMC9441045; doi:10.1186/s12967-022-03607-0)
Supplement: Supplementary file 1 — Additional file 1: Figure S1. The confocal microscopy revealed that HMC3 cells phagocytized exosomes released by U251 and U87 cells. Figure S2. Radiotherapy increased the number of exosomes secreted by glioblastoma multiforme (GBM) cells. A Transmission electron microscopy demonstrated that the secreted exosomes were rounded particles with a double-layer membrane. B The size of these exosomes was approximately 80–100 nm. C Nanoparticle tracking analysis indicated that radiotherapy increased the number of exosomes secreted by U251 and U87 cells. Table S1 All oligonucleotide sequences and Table S2. All primers of RT-PCR. [file 12967_2022_3607_MOESM1_ESM.docx]

**Additional Figure**

**
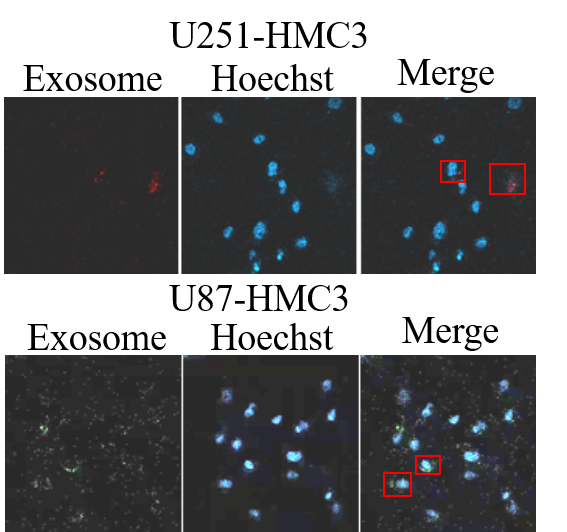
**

**Figure S1.** **The confocal microscopy revealed that HMC3 cells phagocytized exosomes released by U251 and U87 cells.**


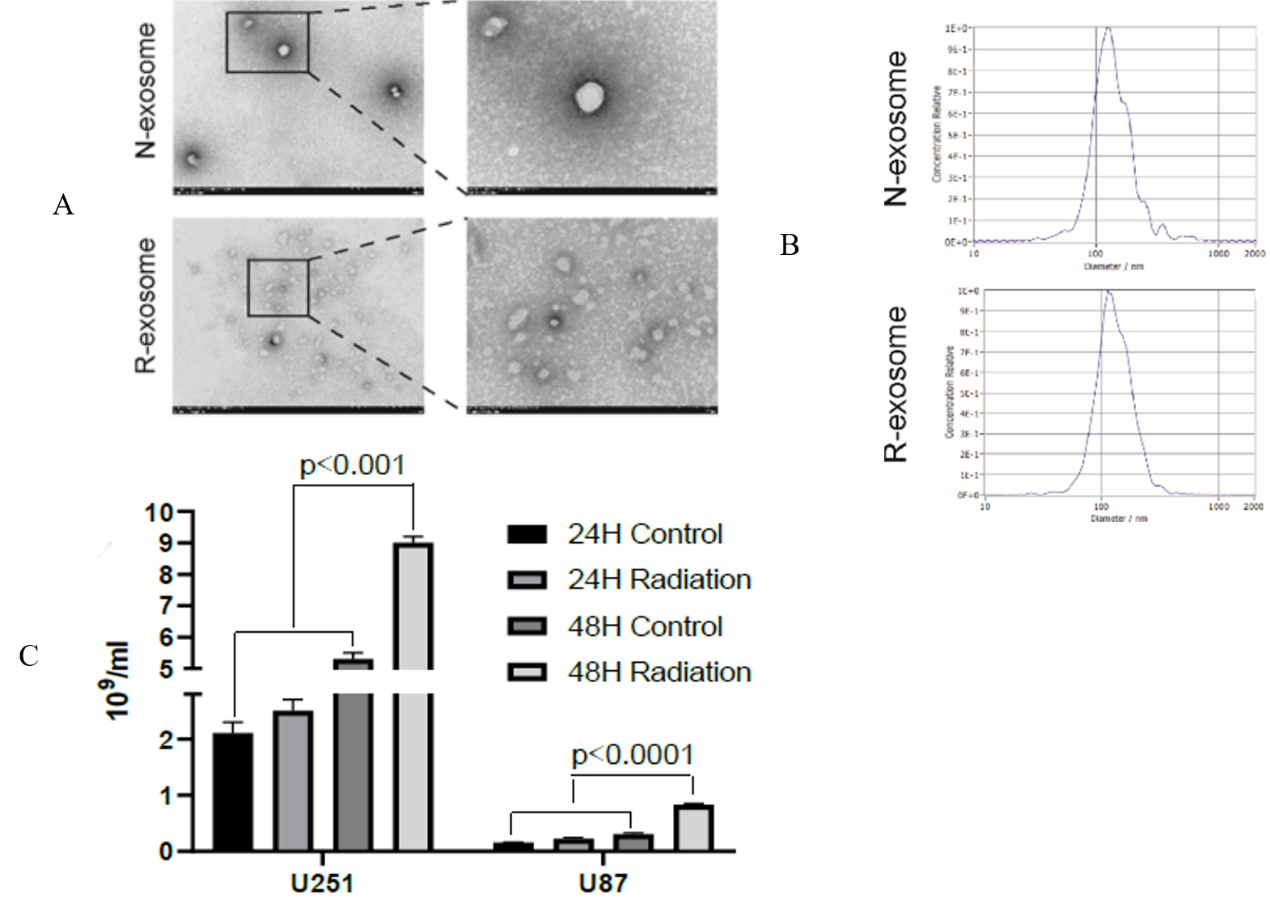


**Figure S2. Radiotherapy increased the number of exosomes secreted by glioblastoma multiforme (GBM) cells. A.** Transmission electron microscopy demonstrated that the secreted exosomes were rounded particles with a double-layer membrane. **B.** The size of these exosomes was approximately 80–100 nm. **C.** Nanoparticle tracking analysis indicated that radiotherapy increased the number of exosomes secreted by U251 and U87 cells.

**Additional Table 1.**

| GENE | SEQUENCE |
| --- | --- |
| mir-340-5p mimics | 5′-UUAUAAAGCAAUGAGACUGAU U-3′  5′-UCAGUCUCAUUGCUUUAUAAUU-3′ |
| miR-NC | 5′-UUCUCCGAACGUGUCACGUTT-3′  5-ACGUGACACGUUCGGAGAATT-3′ |
| anti-miR-340-5p | 5′-AAUCAGUCUCAUUGCUUUAUAA-3′ |
| circ_0012381 | 5`-ATATGGAAATCCAAAGCTTTCAAGAGAAGCTTTGGATTTCCATATTT-3` |
| circ-NC | 5`-GTATGACAACAGCCTCAAGTTCAAGAGACTTGAGGCTGTTGTCATACTT-3` |
| anti-circ_0012381 | 5`-TTCTCCGAACGTGTCACGTTTCAAGAGAACGTGACACGTTCGGAGAATT-3` |

**Additional Table 2.**

| GENE | primer | |
| --- | --- | --- |
| CD163 | forward | 5′-GGCTTGCAGTTTCCTCAAGA-3′ |
|  | reverse | 5′-GACACAGAAATTAGTTCAGCAGCA-3′ |
| IL-10 | forward | 5′-GGCACCCAGTCTGAGAACAG-3′ |
|  | reverse | 5′-TGGCAACCCAGGTAACCCTTA-3′ |
| IL-1 | forward | 5′-TGCTACTTTATGGGCAGCAG-3′ |
|  | reverse | 5′-GGTCGGCAGATCGTCTCTAAA-3′ |
| TGFβ1 | forward | 5′-CTGCAAGTGGACATCAACGG-3′ |
|  | reverse | 5′-TCCGTGGAGCTGAAGCAATA-3′ |
| CCL2 | forward | 5′-TGCAATCAATGCCCCAGTCA-3′ |
|  | reverse | 5′-GGGTCAGCACAGATCTCCTT-3′ |
| TNFα | forward | 5′-CTGCACTTTGGAGTGATCGG-3′ |
|  | reverse | 5′-TCAGCTTGAGGGTTTGCTAC-3′ |
| GAPDH | forward | 5′-GGAGCGAGATCCCTCCAAAAT-3′ |
|  | reverse | 5′-GGCTGTTGTCATACTTCTCATGG-3′ |
| circ_0012381 | forward | 5’-GACTTATAAATATGGAAATCCAAAGC-3’ |
|  | reverse | 5’-AAGTCCCCAGGAAGAGAAGC-3’ |
| miR-340-5p | forward | 5’-CAACCAACCTTATAAAGCAATGAG-3’ |
|  | reverse | 5’-TATGCTTGTTCTCGTCTCTGTGTC-3’ |
| CCR2 | forward | 5ʹ-CCACATCTCGTTCTCGGTTT ATC-3ʹ |
|  | reverse | 5ʹ-CAGGGAGCACCGTA ATCATAATC-3ʹ |
| GAPDH | forward | 5ʹ-TGACTTCAACAGCGACACCCA-3ʹ |
|  | reverse | 5ʹ-CACCCTGTTGCTGTAGCCAAA-3ʹ |
